# Complex Changes in Membrane Lipids Associated with the Modification of Autophagy in *Arabidopsis*

**DOI:** 10.3390/metabo12020190

**Published:** 2022-02-18

**Authors:** Yosia Mugume, Geng Ding, Maria Emilia Dueñas, Meiling Liu, Young-Jin Lee, Basil J. Nikolau, Diane C. Bassham

**Affiliations:** 1 Department of Genetics, Development and Cell Biology, Iowa State University, Ames, IA 50011, USA; ymugume@iastate.edu; 2 Roy J. Carver Department of Biochemistry, Biophysics and Molecular Biology, Iowa State University, Ames, IA 50011, USA; gengding@iastate.edu (G.D.); dimmas@iastate.edu (B.J.N.); 3 Department of Chemistry, Iowa State University, Ames, IA 50011, USA; maria.duenas@newcastle.ac.uk (M.E.D.); yjlee@iastate.edu (Y.-J.L.); 4 Department of Statistics, Iowa State University, Ames, IA 50011, USA; mliu@fredhutch.org; 5 Public Health Sciences Division, Fred Hutchinson Cancer Research Center, Seattle, WA 98109, USA; 6 Center for Metabolic Biology, Iowa State University, Ames, IA 50011, USA

**Keywords:** *Arabidopsis*, autophagy, nitrogen deficiency, MALDI-MSI, lipids, lipidomics

## Abstract

Autophagy is a conserved mechanism among eukaryotes that degrades and recycles cytoplasmic components. Autophagy is known to influence the plant metabolome, including lipid content; however, its impact on the plant lipidome is not fully understood, and most studies have analyzed a single or few mutants defective in autophagy. To gain more insight into the effect of autophagy on lipid concentrations and composition, we quantitatively profiled glycerolipids from multiple *Arabidopsis thaliana* mutants altered in autophagy and compared them with wild-type seedlings under nitrogen replete (+N; normal growth) and nitrogen starvation (−N; autophagy inducing) conditions. Mutants include those in genes of the core autophagy pathway, together with other genes that have been reported to affect autophagy. Using Matrix-Assisted Laser Desorption/Ionization—Mass Spectrometry (MALDI-MS), we imaged the cellular distribution of specific lipids in situ and demonstrated that autophagy and nitrogen treatment did not affect their spatial distribution within *Arabidopsis* seedling leaves. We observed changes, both increases and decreases, in the relative amounts of different lipid species in the mutants compared to WT both in +N and −N conditions, although more changes were seen in −N conditions. The relative amounts of polyunsaturated and very long chain lipids were significantly reduced in autophagy-disrupted mutants compared to WT plants. Collectively, our results provide additional evidence that autophagy affects plant lipid content and that autophagy likely affects lipid properties such as chain length and unsaturation.

## 1. Introduction

Autophagy is a pathway for the vacuolar degradation and recycling of cellular organelles and macromolecules. It is activated as a developmental process, as a housekeeping mechanism, or in response to stress. It is conserved among eukaryotic organisms, and macroautophagy, referred to here as autophagy, is the best-studied form of autophagy [1,2,3]. In plants, a basal level of autophagy during growth and development ensures homeostasis by degrading obsolete cytoplasmic materials and recycling the breakdown products. Autophagy is upregulated under environmental stresses to aid plant survival through the catabolism and recycling of damaged organelles and the elimination of toxic protein aggregates. It is also upregulated by developmental cellular reprogramming processes such as senescence [4,5,6], xylem development [7], and by temporary cellular reprogramming triggered by phytohormones, immune response, and danger signals [8]. Autophagy is hallmarked by the de novo formation of double membrane vesicles known as autophagosomes, which are formed in the cytoplasm through the action of autophagy-related (ATG) proteins. Autophagosomes encapsulate and sequester unwanted cytoplasmic constituents, transporting them to the lytic vacuole. The autophagosome contents are then degraded by proteases and other hydrolases, and recycled for reuse by the cell [2,3].

In yeast (*Saccharomyces cerevisiae*), over 40 ATG proteins that drive the autophagic process have been described, and orthologs of most of these proteins have also been identified in plants [3]. These proteins hierarchically orchestrate the autophagy pathway from induction, vesicle nucleation, phagophore membrane expansion and closure, delivery of the autophagosome to the vacuole, and, finally, the breakdown of the autophagic membrane and digestion of its contents [3].

Autophagy is regulated by Target of Rapamycin (TOR), a serine/threonine kinase, that is part of the TOR complex (TORC). The TORC negatively regulates autophagy in plants, potentially by phosphorylating ATG13, a component of the initiation complex, thus preventing its association with ATG1, while the rapid dephosphorylation of ATG13 upon TORC inactivation allows ATG1 binding [9,10]. Sucrose Non-Fermenting1–Related Kinase 1 (SnRK1) also regulates autophagy upstream of the TORC. KIN10 is the SnRK1 catalytic subunit and the *kin10* mutant is unable to induce autophagy during stress [11]. RNS2, a vacuolar endoribonuclease of the RNase T2 family, also influences autophagy through TORC signaling, and *rns2* mutants have constitutive autophagy [12,13].

Autophagy is integrated with lipid metabolism in plants and algae [14]. In *Chlamydomonas reinhardtii*, autophagy plays a role in triacylglycerol (TAG) biosynthesis and the formation of lipid droplets (LD), which store TAG under stress conditions [15]. In *Arabidopsis thaliana*, autophagy contributes to TAG synthesis via the turnover of organellar membranes under normal growth conditions [16]. On the other hand, autophagy operates in the degradation of LDs by lipophagy, a selective microautophagy mechanism that involves the direct engulfment of LDs via tonoplast invagination under extended darkness [16]. These observations are consistent with those in maize (*Zea mays*), where under N starvation, an autophagy-defective *atg12* mutant accumulates LDs, which could reflect the disruption of LD catabolism or active LD assembly [17].

In addition to being substrates for autophagy, specific lipids have been shown to be important for autophagosome biogenesis. For example, phosphatidylinositol (PI) is phosphorylated by PI3-kinase to generate PI3P on the target membrane, which is critical for autophagy [18]. Phosphatidylethanolamine (PE) is conjugated to ATG8 during autophagosome elongation, thus enabling its incorporation into the growing autophagosome membrane [1,19]. Analyses of autophagosomes from human cells, yeast, and *Drosophila* identified several phospholipids in different proportions as potential constituents of autophagosome membranes [20,21,22]. The source of lipids used for the formation of autophagosomes is still incompletely understood, and in mammals and yeast may include the ER, Golgi, endosomes, mitochondria, plasma membrane, and lipid droplets [23]. Recent studies showed that ATG2 mediates the transfer of lipids from the ER to expanding phagophores in yeast and mammals [24,25]. Phospholipids can also be synthesized de novo to drive autophagic membrane expansion in yeast cells [20]. In plants, the autophagosomal membrane is also associated with the ER [26,27], suggesting that the ER provides lipids for phagophore expansion in plants as well.

Recent studies have shown the involvement of autophagy in lipid metabolism in plants, revealing altered lipid amounts in *atg* mutants compared with WT plants, both under stress and non-stress conditions [16,28,29,30,31]. Most previous work has analyzed a single or small number of autophagy mutants, and whether mutants with increased autophagy have changes in lipid content has not been addressed. To gain more insight into the influence of autophagy on lipid metabolism, we assessed the lipidome of an array of *Arabidopsis* mutants altered in autophagy, grown under nitrogen replete (+N) and nitrogen starvation (−N) conditions, including mutants that are blocked in autophagy activation, and those with increased basal autophagy (Table 1). We observed an impact of autophagy level on all lipid classes analyzed, with some lipids showing a decrease while others showed an increase in *atg* mutants compared with the WT control. We also observed changes in unsaturation and acyl chain length in autophagy mutants compared to WT, thus strengthening the hypothesis that autophagy impacts lipid metabolism. Additionally, we imaged lipids in situ using Matrix-Assisted Laser Desorption/Ionization—Mass Spectrometry (MALDI-MS), which revealed that there are no detectable changes in the spatial distribution of lipids across leaves of all the genotypes evaluated.

## 2. Results

### 2.1. Nitrogen Starvation Induces Autophagy and Causes Changes in the Lipidome

To explore the impact of autophagy on lipids in *Arabidopsis*, we carried out a lipidomic analysis on seedlings from nine different genotypes, with growth occurring in nitrogen replete (+N; control) and in nitrogen deplete (−N; autophagy-inducing) conditions [33] (Table 1). This experimental design allowed us to distinguish between true effects of changes in autophagy from changes associated with N-starvation, as well as any potential additional functions of ATG proteins that are unrelated to autophagy. The genotypes studied included wild-type (accession Columbia-0) and the autophagy machinery mutants a*tg5-1*, *atg7-2,* and *atg9-4*, hereafter labelled as *atg5*, *atg7,* and *atg9*; mutants in non-*ATG* genes that affect autophagy activity included *kin10* (unable to induce autophagy), *raptor1b,* and *rns2-2*, hereafter labelled as *rns2*, (constitutive autophagy); and double mutants *rns2*; *atg5* and *rns2*; *atg9*.

A direct infusion electrospray ionization-triple quadrupole mass spectrometry (ESI-MS/MS) analysis of lipid extracts identified 120 lipid species, including the glycolipids monogalactosyldiacylglycerol (MGDG) and digalactosyldiacylglycerol (DGDG), and the phospholipids phosphatidylglycerol (PG), phosphatidylcholine (PC), phosphatidylethanolamine (PE), phosphatidic acid (PA), phosphatidylserine (PS), lysophosphatidylcholine (LPC), lysophosphatidylglycerol (LPG), and phosphatidylinositol (PI) (Supplementary Materials Table S1).

**Figure 1:**
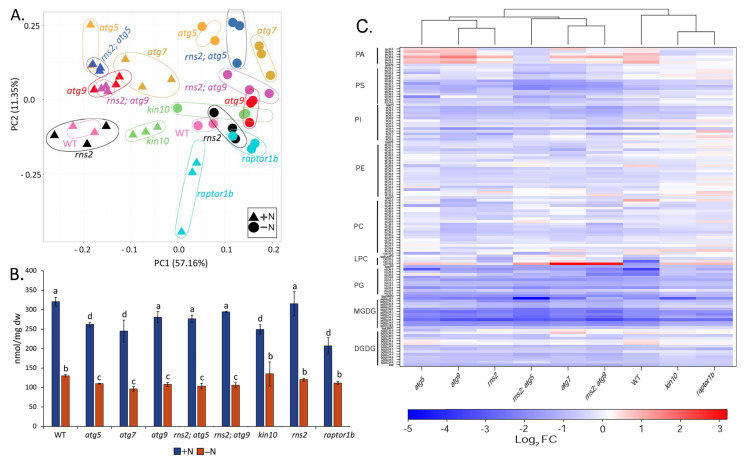
Nitrogen treatment and genotype both influence the plant lipidome. (**A**) Principal component analysis (PCA) for autophagy-blocked mutants (*atg5*, *atg7*, *atg9*, *rns2*; *atg5, rns2*; *atg9*, and *kin10*), constitutive autophagy mutants (*rns2* and *raptor1b*), and WT. The values were determined from log10-transformed lipidomics data (*n* = 3 biologically independent samples; for WT and *atg5*, *n* = 2 biologically independent samples). The amounts of variation explained by the first two components are indicated on the axes. The dashed circles indicate biological replicates for each genotype/condition. (**B**) Total membrane lipid amount in *Arabidopsis* seedlings. Total lipid amount represents the sum of the amount of all lipid species measured from each of the genotypes under + and -N. Values (lipid/dry weight (nmol/mg)) are means ± standard deviation (SD), *n* = 3 biologically independent samples and for WT and *atg5*, *n* = 2 biologically independent samples. Different letters denote statistically significant differences between means (q < 0.1). (**C**) Nitrogen starvation causes a reduction in most lipid species. Log_2_ Fold Change (FC) heat map of lipid species for each genotype under −N versus +N comparison. Blue indicates a reduction and red indicates an increase in lipids. Log_2_ FC values were calculated from data presented in Supplementary Materials Table S2.

A principal component analysis (PCA) based on the lipid profile data showed that most of the variance was included in the first two principal components, explaining about 70% of the overall data variance (56% for PC1 explaining the N-treatment effect and 11% for PC2 explaining the genotype effect) (Figure 1A). This shows that both N-treatment and genotype had an effect on the lipid content of the seedlings, with the N-treatment effect explaining most of the variation observed. In +N conditions, all genotypes clustered closely together, except for *raptor1b*. In −N conditions, which activates autophagy, separation was seen between genotypes, with autophagy-blocked mutants (*atg7*, *atg5*, *atg9*, *kin10*, *rns2*; *atg5,* and *rns2*; *atg9*) clustering together, and mutants with constitutive autophagy (*rns2* and *raptor1b*) clustering with WT (Figure 1A). The clustering pattern is therefore consistent with the extent of autophagy under different conditions and reinforces the observation that autophagy activity affects the lipid profile of seedlings.

The total amount of the measured lipids was significantly reduced in all genotypes in the −N condition. In the +N condition, the *atg5*, *atg7*, *kin10*, and *raptor1b* mutants showed a significant reduction in total lipid amount as compared to the WT control. Given that *raptor1b* shows constitutive autophagy, this observation reveals a complex relationship between autophagy activity and lipid content, and could reflect the enhanced catabolism of lipids by autophagy or disruption of active lipid assembly. Under −N conditions, *atg5*, *atg7, atg9, rns2*; *atg5*, and *rns2*; *atg9* mutants showed a significant reduction in total lipid amounts as compared to WT plants, consistent with all of these mutants being deficient in autophagy, while *kin10*, *rns2*, and *raptor1b* mutants showed no significant difference compared to WT (Figure 1B,C). Consistent with a previous study [37], the proportion of MGDG was reduced in −N conditions, with a concomitant increase in other lipids (Supplementary Materials Figure S1).

A heat map representation of the log_2_ fold change in the amount of individual lipid species in response to N starvation shows that most lipid species are significantly reduced across all genotypes (Figure 1C). The *raptor1b* and *kin10* mutants have the fewest number of lipid species significantly changed by N starvation (Figure 1C). Unlike other lipids, which generally decrease in −N, lysophospholipids increased in accumulation in a number of genotypes. Because lysophospholipids are primarily products of phospholipases, this finding is consistent with previous reports that showed an increased accumulation of these lipid breakdown products during carbon starvation [31] and freezing stress [38] in *Arabidopsis*, and under N starvation in maize [28]. The amounts of most PA species were significantly increased upon nitrogen starvation (Figure 1C). This is consistent with previous studies in *Arabidopsis*, which showed that in response to N starvation, phospholipids are hydrolyzed by phospholipase D ε to generate PA [39]. Together, these results show that N treatment affects both the quantity and composition of lipids.

### 2.2. Matrix-Assisted Laser Desorption/Ionization—Mass Spectrometry (MALDI-MS) Imaging of Lipids in Arabidopsis under + and −N Conditions

Mass spectrometry imaging (MSI) has become a valuable tool for analyzing the spatial distributions of a wide range of metabolites in situ [40,41,42,43,44,45,46,47], including in plants [44,46,47,48]. We therefore used MALDI-MS to image lipids in situ and to determine the effect of autophagy and −N or +N conditions on their spatial localization across a leaf in *Arabidopsis*.

We used a leaf fracture method, which has been demonstrated to maintain the original leaf anatomy [49], and imaged the distribution of lipids within the leaves. A leaf was “split” into two halves parallel to the plane of the leaf, and the two exposed leaf interior surfaces were imaged visually and by MALDI-MS. The resulting two images were then aligned and compared to identify the distribution of molecular features with respect to visually identified features. Lipid distributions were imaged in fractured leaves from several genotypes (WT, *atg5*, *atg9*, *atg7*, *rns2*, *rns2*; *atg5*, and *rns2*; *atg9*) that had been grown either in +N or −N conditions.

**Figure 2:**
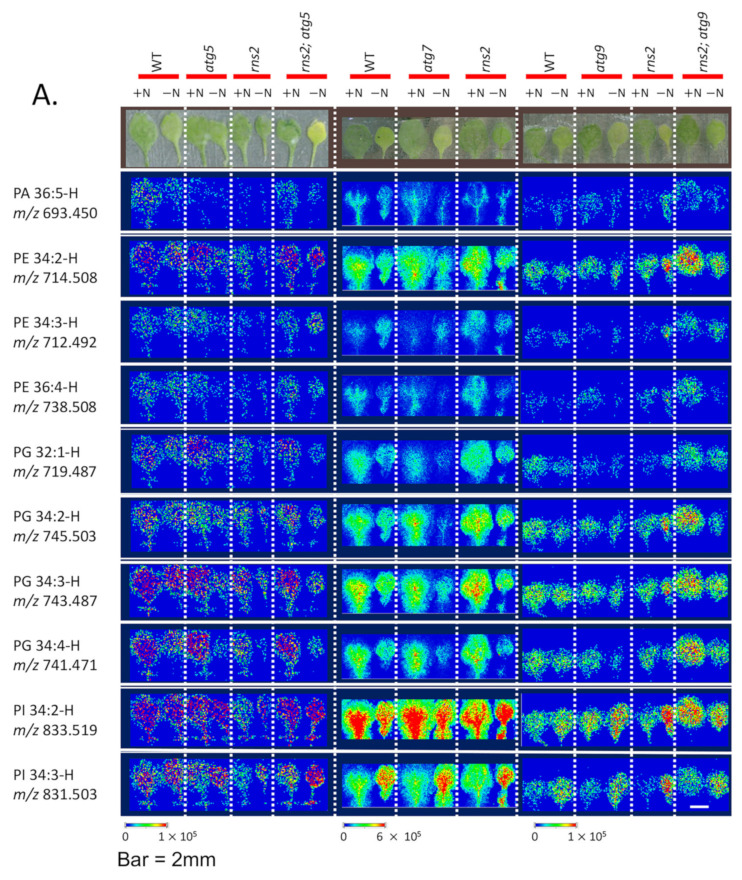

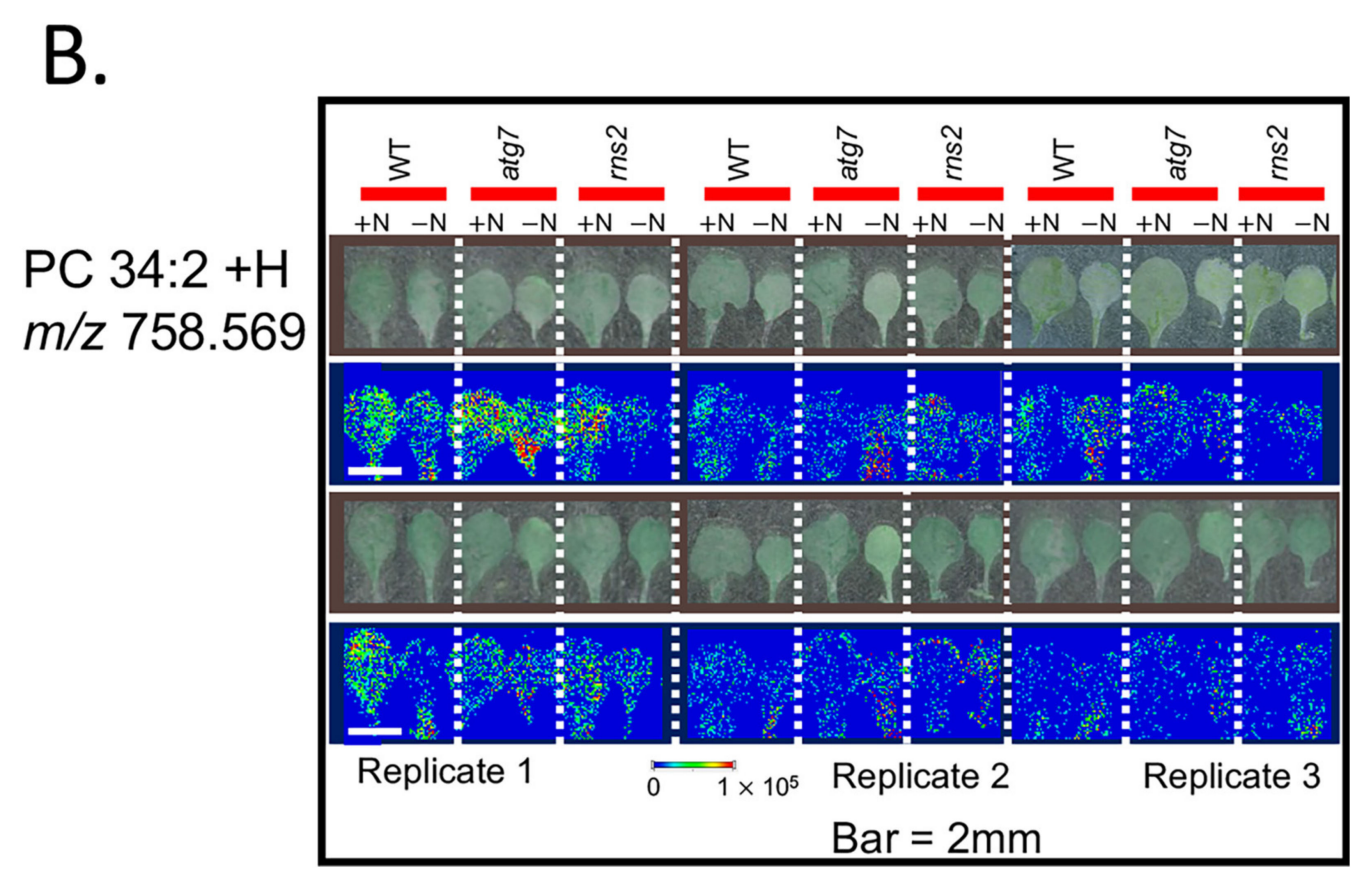
Mass spectrometry images of lipid species in each half of fractured wild-type and mutant *Arabidopsis* leaves. (**A**) 100-micron resolution MS images of the representative lipid species indicated at the left, imaged in negative mode, in WT and the indicated mutant genotypes grown in +N or −N conditions. (**B**). Images of PC 34:2, chosen as a representative lipid, imaged in 3 replicates in different genotypes in positive mode, showing reproducibility in spatial distribution between replicates. In all cases, both halves of the fractured leaves are shown. The scale bar shows the relative intensities within a sample.

Based on the ESI-MS lipidomics data, we selected specific lipid molecules to image, which showed significant changes in abundance in the −N vs. +N comparisons (i.e., PE34:2, PE34:3, PE36:4, PG32:1, PG34:2, PG34:3, PG34:2, PG34:4, PI34:2, and PI34:3); additionally, we imaged PA36:5, which showed no significant change in abundance as measured by ESI-MS. These lipid species were found to be uniformly localized throughout the exposed interior leaf surfaces examined (Figure 2A). Moreover, this uniform distribution was unaffected by any of the mutations that we studied, nor was it affected by the N-treatment that was applied to these plants, both of which affect autophagy. Note that due to analytical and biological variation between samples, direct comparisons should be made only in reference to each corresponding wild-type sample within a single experimental replicate and cannot be made between experiments. These findings, therefore, indicate that the lipid metabolism response to changes in autophagic status are triggered simultaneously among all the cells visible throughout the leaf (Figure 2A), which may indicate that autophagy is active in all of the cells observed in the leaves. While different signal intensities are observed between replicates, the spatial distribution of the signal within an individual leaf is reproducible, as illustrated in Figure 2B, which shows replicate images of independent biological replicates of each half of a leaf for PC34:2.

### 2.3. Autophagy Alters the Amount of Specific Lipid Species

Given that N starvation is known to induce autophagy [11,33,36], we examined whether the observed changes in lipid amounts in response to N conditions are dependent on autophagy. Lipids with different polar head groups have been shown to be important for the process of autophagy [50,51] and also as cargo degraded/remobilized by autophagy [16]. Based on this, we profiled the impact of autophagy on levels of polar membrane lipids. We compared changes in lipid amounts between WT plants and each of the mutants under control (+N) and autophagy-inducing conditions (−N).

**Figure 3:**
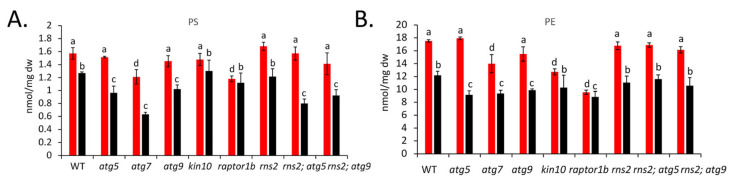
Changes in selected autophagy-related lipids in different N conditions. Total PS (**A**) and PE (**B**) was measured in +N (red) and −N (black) conditions in the indicated genotypes. Values (nmol lipid/ mg dry weight) are means ± standard deviation (SD), (*n* = 3 biologically independent samples; for WT and *atg5*, *n* = 2 biologically independent samples). Different letters denote statistically significant differences between means within each N-treatment (q < 0.1).

**Figure 4:**
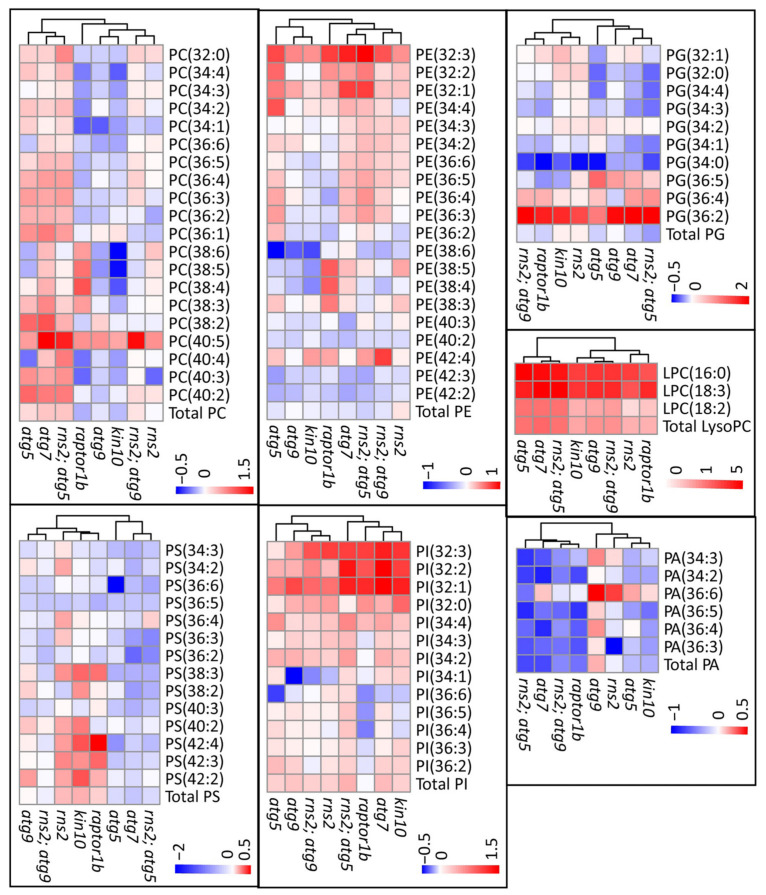
Lipid changes in autophagy-altered mutants. Log_2_ fold changes between mutants and WT plants are shown under −N conditions as heat maps with shades of red (increased) or blue (decreased) colors depicting changes according to the scale bar. The lipids represented on the heat maps are phosphatidylethanolamine (PE), phosphatidylcholine (PC), phosphatidylinositol (PI), phosphatidylglycerol (PG), phosphatidylserine (PS), lysophosphatidycholine (LPC), and phosphatidic acid (PA), with the chain length and number of double bonds of individual lipid species in parentheses.

An analysis of lipid amounts revealed differences between WT and autophagy-related mutants, both under +N and −N conditions. As an example, in −N (autophagy inducing) conditions, total PS was significantly reduced in all autophagy-blocked mutants except for *kin10*, while the constitutive autophagy mutants *raptor1b* and *rns2* showed no significant change when compared with the WT control. Under +N conditions, only the *raptor1b* mutant showed a significant reduction in levels of PS as compared to WT (Figure 3 and Figure 4). Total PE was decreased in *atg5*, *atg7*, *atg9*, and *raptor1b* mutants under −N, while under +N, it was significantly reduced in *atg7*, *kin10*, and *raptor 1b*. Overall, in conditions in which autophagy is active in WT plants, a reduction in the amount of PS and PE is seen in autophagy-deficient mutants. *raptor1b* and *kin10* seem to behave differently to other mutants, possibly reflecting their roles in many cellular pathways in addition to autophagy, or as regulators rather than core components of the pathway.

We assessed the impact of changes in autophagy on individual lipid species within the major lipid groups. Under −N conditions, most PS molecular species were significantly reduced in autophagy-blocked mutants (*atg5*, *atg7*, and *rns2*; *atg5*), while no significant change was observed for most of the PS molecular species in *kin10*, *rns2*, and *raptor1b* (Figure 4). Very few PS species showed significant changes in their amount compared to WT under +N conditions (Supplementary Materials Figure S2). Changes in PE species were less consistent, with some species increased and some decreased.

Several PC and PI lipid species were also increased in autophagy-altered mutants, irrespective of +N or −N conditions. Interestingly, PC34:4, PC34:3, PC34:2, PC34:1, PC36:4, PC36:2, PI34:2, and PI34:3 were significantly increased in autophagy-blocked mutants compared to WT, but not in the constitutive autophagy mutants, *raptor1b* and *rns2* (Figure 4). One possibility is that these lipids are preferentially degraded by autophagy, thereby explaining their accumulation in *atg* mutants.

An increase in lysophosphatidylcholine (LysoPC) concentration was detected in all *atg* mutants compared to WT in +N. Under −N conditions, LysoPC was increased compared to WT in all genotypes tested (Figure 4). This is consistent with previous studies that showed an increased accumulation of lysophospholipids in plants under different stresses [28,31,38]. Because lysolipids are considered as breakdown products of glycerol lipids, these findings suggest an increase in lipolytic activity in mutants compared to WT plants in response to N starvation. Other lipid species measured (PA and PG) showed fewer differences between genotypes in response to nitrogen conditions (Figure 4).

Together, these results show that some lipids decrease in the *atg* autophagy-blocked mutants, while others increase in these mutants when plants are grown under −N conditions. One possibility is that some of the lipids are incorporated into the autophagosome membrane, and thus the activation of autophagy triggers an increase in the biosynthesis of such lipids to meet the demand for autophagosome formation. An increase in some lipids in the *atg* mutants compared to WT could be due to the degradation of these lipids in WT plants by autophagy, which is lost in in *atg* mutants.

### 2.4. Lipids with Longer Chain Unsaturated Fatty Acids Are Decreased in Autophagy Mutants in Response to N Starvation

**Figure 5:**
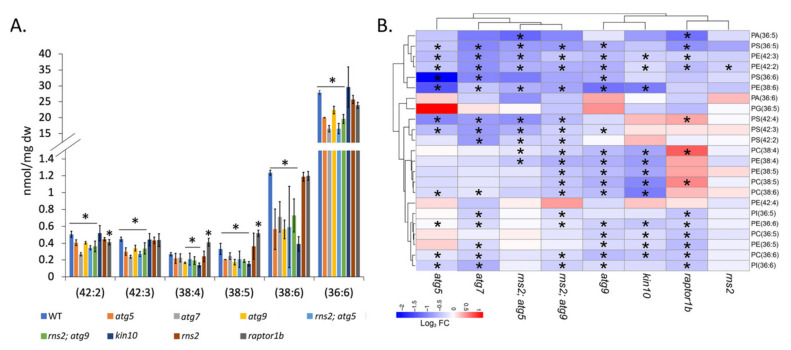
Polyunsaturated and very long chain fatty acids are significantly reduced in autophagy-blocked mutants compared to WT plants in response to N starvation (**A**). Changes in lipid amounts among lipid groups. The bar graph represents the amount of lipids per group based on acyl chain length and the number of double bonds. Groups represent a summation of all lipid species from all polar head groups with the same chain length and number of double bonds. Shown are the means ± SD (*n* = 3 biologically independent samples; for WT and *atg5*, *n* = 2 biologically independent samples). * denotes statistically significant differences between means (q < 0.1). (**B**) Heat map depicting log_2_ fold changes in individual lipids that make up the lipid groups in mutants compared with WT plants under −N conditions, with shades of red (increased) or blue (decreased) colors depicting changes according to the scale bar. * denotes statistically significant differences (q < 0.1). Clustering for heat maps was performed using the pheatmap package in R (https://cran.r-project.org/web/packages/pheatmap/pheatmap.pdf (accessed on 14 February 2022)).

In the autophagy-blocked mutants grown under −N conditions, we found a significant reduction in lipids with longer chain fatty acids and in lipids with higher levels of acyl chain unsaturation (i.e., 36:6, 38:4, 38:5, 38:6, 42:2, and 42:3 lipids). This alteration in lipid accumulation is not apparent in the autophagy-constitutive mutants (Figure 5A). Except for 36:6, no difference in these lipids was seen in the mutants under N-replete conditions. Given that these changes are observed in the −N condition that induces autophagy, but disappear in the +N condition, they are most likely dependent on autophagy. Additionally, we looked at changes in the relative amount for individual lipid species from the groups identified in Figure 5A; we observed a significant decrease in representative individual lipid species in autophagy-blocked mutants compared to WT plants under −N conditions, with minimal changes in constitutive autophagy mutants (Figure 5B). Most of the significant changes in the individual lipid species are diminished under +N conditions (Supplementary Materials Figure S3).

### 2.5. Autophagy Induction Alters Lipid Unsaturation

**Figure 6:**
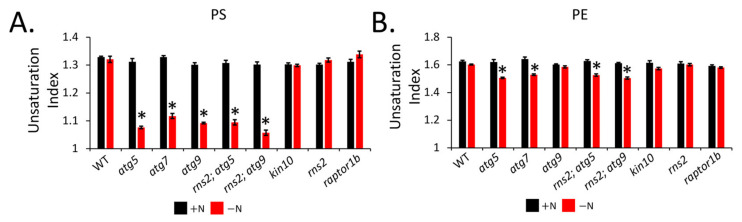
The unsaturation index of PS (**A**) and PE (**B**) is decreased in *atg* mutants compared to WT plants grown under −N conditions. The unsaturation index of each lipid molecular species was calculated, and the unsaturation indices of individual lipid molecular species summed to give the total unsaturation index of a lipid. Values shown are means ± SE; *n* = 3, *p* < 0.05 is indicated by *.

Lipid groups and individual lipid species containing polyunsaturated fatty acids were decreased in autophagy mutants compared to WT seedlings (Figure 6). We hypothesized that the induction of autophagy may lead to decreased lipid saturation. To test this hypothesis, we measured the total unsaturation index of lipid species, defined as the product of the amount of that species and the average number of double bonds per acyl chain [52,53]. Under N-replete conditions, there was no difference in the unsaturation index for any lipid in any of the genotypes. However, under N starvation, PE and PS both showed a decrease in the unsaturation index in autophagy mutants compared to the WT control. Other lipids, such as MGDG, DGDG, PI, PG, PC, and PA, showed no change in the unsaturation index across all genotypes under −N conditions (not shown).

## 3. Discussion

During autophagy, lipids can be both cargoes, and constituents, of autophagosome membranes. Several studies have recently reported on the alterations of lipid content in autophagy mutants under different stress conditions in plants. However, lipidomic changes in distinct autophagy mutants do not show a complete overlap [28,30,31], potentially because these mutants affect different steps in the autophagy pathway, allelic differences give rise to strong and weak mutant phenotypes [28], or *ATG* genes may function in other processes in addition to autophagy [54]. We reasoned that a lipidomic analysis of a wider range of mutants would help to pinpoint the specific effects of autophagy on lipid metabolism in *Arabidopsis*.

We used ESI-MS to quantitatively profile lipids and MALDI-MS to image lipids in situ from mutants affecting the core autophagy pathway (*atg5*, *atg7*, and *atg9*), mutants that do not affect the core autophagy machinery but that affect autophagy activity (*kin10*, *raptor1b*, and *rns2*) and double mutants (*rns2*; *atg5* and *rns2*; *atg9*) to characterize changes in membrane lipid amounts in response to autophagy.

### 3.1. Autophagy Affects Lipid Concentrations under Both +N and −N Conditions

We observed that the *raptor1b* mutant clustered away from other genotypes in a PCA and has a significant reduction in total lipids compared to the WT control under +N conditions, but not under −N conditions. This suggests that the *raptor1b* lipidome is significantly altered, even under non-stress conditions. RAPTOR1B, a conserved TOR complex subunit, is a scaffold protein which recruits substrates for the TOR kinase. *raptor1b* mutants disrupt TOR signaling and exhibit molecular and physiological phenotypes of TOR inhibition [36,55].

TOR activation is reported to upregulate the expression of lipid synthesis genes and downregulate lipid degradation genes in *Arabidopsis* [56], as well as galactolipid synthesis genes in rice [57]. Thus, the reduction in total lipids in *raptor1b* could be due to reduced lipid synthesis. TOR also negatively regulates autophagy [58,59], and autophagy has been implicated in TAG synthesis and lipid droplet accumulation, likely from the breakdown of membrane phospholipids [15,16]. Since *raptor1b* has constitutive autophagy [36], lipid breakdown by autophagy may also contribute to the decrease in the total lipid amount in *raptor1b* compared to WT under +N conditions. However, *rns2,* which also has constitutive autophagy [12], behaved differently from *raptor1b*, showing no significant change compared to WT with regard to total lipid amounts, and also clustered closer to WT in the PCA under both +N and −N conditions. This suggests that changes in lipid synthesis may be a more significant factor affecting lipids in *raptor1b* rather than autophagic degradation.

The autophagy mutants *atg5* and *atg7* had significantly less total lipid than the WT control under +N conditions, while the *atg9* mutant showed no significant change. The discrepancy in the *atg9* result is probably due to the known weaker autophagy phenotype of *atg9* [30,60]. It is not clear whether the observed significant reduction in total lipid amounts in *atg5* and *atg7* compared to WT is attributable to reduced lipid biosynthesis or increased degradation in these mutants. In contrast to our observation, a study by Fan et al., 2019 showed that a disruption of autophagy results in a decrease in membrane lipid turnover under non-stress conditions [16]. The differences in the systems used in our study (*Arabidopsis* seedlings) and Fan et al., 2019 (mature and senescing leaves) may explain this discrepancy. One possibility is that a disruption of autophagy results in the activation of other lipid catabolic pathways, therefore increasing lipid breakdown in autophagy mutants even under non-stress conditions. Consistent with this hypothesis, a transcriptomics analysis of maize plants undergoing N starvation revealed the upregulation of phospholipases in *atg* mutants when compared to WT plants [17].

*kin10* showed a significant reduction in total lipids compared to WT under +N, and no significant change under −N conditions, an unexpected observation as this mutant has similar levels of autophagy as WT plants under +N conditions [11]. KIN10 is the catalytic subunit of *Arabidopsis* SnRK1 and regulates autophagy upstream of TOR. As KIN10 activates lipid catabolism genes and inhibits lipid biosynthesis genes [61,62], the reason for the observed changes in lipid content remains obscure and requires further investigation.

### 3.2. MALDI-MS Imaging of Lipids

Modern advances in mass spectrometry have made it possible to quantitatively profile acyl lipids, and have thus improved our understanding of lipid metabolism in plants [40,63,64,65,66,67,68]. MS lipid profiling has mostly been performed through the extraction of pulverized tissue, yet it has recently been shown that lipids undergo dynamic spatial changes in plant tissues during development [69]. Therefore, we used MALDI-MS to image lipids in situ and assess the effect of autophagy and N-treatment on spatial lipid localization across the leaf in *Arabidopsis*. Our results show that neither N starvation, nor autophagy, impacted the spatial distribution of lipids in the seedlings, with changes in lipid profiles occurring evenly throughout the leaf, at least in the mesophyll cells that are imaged by this technique.

### 3.3. Autophagy Mutants Have Increases or Decreases in Different Lipid Species

The relative amount of some lipid species was increased, while others decreased in *atg* mutants compared to the WT control under −N conditions, either due to changes in lipid synthesis or degradation. Autophagy is marked by the formation of an autophagosome, a double membrane vesicle that carries cargo to the vacuole. A number of lipids are important for autophagosome formation and as constituents of autophagosome membranes [23,70]; for example, PE, which is conjugated to the key autophagy protein ATG8. We have observed changes in the amounts of some of these key lipid species in response to N depletion or in autophagy mutants. Studies in yeast and human cells have demonstrated the de novo synthesis of lipids for autophagosome formation [20,71]. Therefore, it is likely that lipids are synthesized in WT seedlings to meet the demand for autophagosome formation, but whether this synthesis is impaired in autophagy mutants is not clear. The observation that LPC, a breakdown product of PC, is increased in all autophagy-blocked mutants compared to WT under −N conditions is consistent with the proposal by McLoughlin et al., 2018 that autophagy might be essential for recycling membrane lipid turnover products [28].

### 3.4. Unsaturated and Long Chain FAs Are Reduced in Autophagy Mutants

Acyl chain length and unsaturation are fatty acid properties that have been reported to be impacted by autophagy [20,21,22,29,71,72,73,74]. We, therefore, reasoned that the induction of autophagy may affect the saturation and carbon chain length of lipid fatty acyl chains in *Arabidopsis*. To this end, we looked at changes in the amount of lipids based on acyl chain length and the total number of double bonds. We observed a decrease in the amounts of lipids containing unsaturated FAs and with long acyl chains in autophagy-blocked mutants compared with the WT control. This observation is consistent with a previous study in rice that showed a decrease in unsaturated lipids extracted from anthers of *Osatg7* compared to WT [75]. Lipidomic analysis of purified autophagosomes and autophagic membranes revealed an enrichment of unsaturated lipids in yeast and *Drosophila* [21,22], and defects in lipid desaturation resulted in autophagic defects [76]. This suggests that lipid desaturation may be important in autophagy.

VLCFAs are increased in plants under various abiotic constraints [77]. However, our observation that they are reduced in *atg* mutants compared to WT plants shows that LCFAs and VLCFAs might be important for autophagy. In contrast to our results, an increase in long acyl chains in *atg5* compared with WT was previously reported [29]; that study included sphingolipids, potentially explaining this difference.

It is likely that changes in lipid species saturation and chain length could modify the physical properties of membranes, such as thickness, fluidity, and membrane curvature, which could be important for autophagosome formation. For example, the introduction of double bonds and reduced acyl chain length increase the membrane fluidity and flexibility, while increased fatty acyl chain length leads to thicker bilayer structures [78]. The appropriate balance of various phospholipid species might ensure the ideal physical properties for the membrane dynamics required when autophagy is induced.

In conclusion, by using multiple mutants, we show that autophagy has an impact on lipid content and affects lipid unsaturation and acyl chain length properties. A decrease in the amount of total lipids was seen under −N conditions for all genotypes, but this decrease was greater for mutants blocked in autophagy. Significant changes in individual lipid species were seen between mutants and WT plants, both in +N and −N conditions, but again, a greater number and more substantial changes were seen under −N conditions; this was particularly evident for PS and PE. Mutants blocked in autophagy also had significantly reduced relative amounts of polyunsaturated and very long chain lipids when compared with WT. Our data suggests an effect of autophagy on glycerol lipid degradation pathways, but also a potential role for autophagy in promoting the biosynthesis of these lipids.

## 4. Materials and Methods

### 4.1. Plant Materials, Growth, and Nitrogen Treatment

*Arabidopsis thaliana* genotypes used are WT (Col-0); T-DNA insertion mutants *rns2-2* (SALK_069588) [79], *atg9-4* (SALK_145980) [12], *atg5-1* [80], *atg7-2* [32], *kin10* (SALK_127939C) [81,82], *raptor1b* (SALK_078159) [83]; and double mutants *rns2-2 atg5-1* and *rns2-2 atg9-4* [12]. *Arabidopsis* seeds were sterilized with 33% (*v*/*v*) bleach and 0.1% (*v*/*v*) Triton X-100 (Sigma,- St Louis, MO, USA) for 20 min, followed by five washes of 5 min each with sterile water. Sterilized seeds were kept at 4 °C in darkness for at least 2 days to allow for stratification, before being plating on solid ½-strength MS medium (2.22 g/L Murashige–Skoog vitamin and salt mixture (Caisson Laboratory), 1% (*w*/*v*) sucrose, 0.6% (*w*/*v*) Phytoblend agar (Caisson Laboratory), and 2.4 mM 2-morphinolino-ethanesulfonic acid (MES, Sigma), pH 5.7). Seedlings were grown under long-day conditions (16 h light) at 22 °C for 7 days. For nitrogen starvation, 7-day-old seedlings grown on solid ½ MS medium were transferred to solid ½ MS medium lacking nitrogen for an additional 3 days, while the control seedlings were also transferred to solid ½ MS medium [33].

### 4.2. Lipid Extraction

Lipid extraction was carried out as previously described [68]. Seedling samples were immediately dropped into 3 mL of isopropanol with 0.01% butylated hydroxytoluene (BHT) preheated to 75 °C in a 50 mL glass tube with a Teflon lined screwcap (Thermo Fisher Scientific, Inc., Waltham, MA, USA). The sample-containing tubes were maintained at 75 °C for 15 min to deactivate lipid-hydrolyzing enzymes such as phospholipase D. Next, 1.5 mL of chloroform and 0.6 mL of water were added, followed by shaking on an agitating shaker for 1 h. Extracted lipids (first extract) were transferred to a new tube using a Pasteur pipette, leaving the seedling debris at the bottom of the original tube. Following the first extraction, 4 mL of chloroform: methanol (2:1) with 0.01% BHT were added to the seedling debris, and the samples were shaken on an orbital shaker at room temperature for 30 min, with the solvent then being transferred to the first extract. This was repeated 3 times, and for the 4th time, the samples were shaken on an orbital shaker at room temperature overnight while the combined extract was kept at −80 °C. After overnight shaking, the solvent was transferred to the first extract, and, at this stage, all of the seedling debris appeared white. The solvent was evaporated from the extract in an N-EVAP 112 nitrogen evaporator (Organomation Associates, Inc., Berlin, MA, USA). The lipid extract was dissolved in 1 mL of chloroform, transferred to a 2 mL clear glass vial with a Teflon-lined screw cap (DWK Life Sciences L.L.C., Millville, NJ, USA), and stored at −80 °C. Prior to shipping, the solvent (chloroform) was again evaporated from the lipid extract in the N-EVAP 112 nitrogen evaporator. The vials containing the lipid extracts were shipped overnight to the Kansas Lipidomics Research Center (KLRC) on dry ice. At KLRC, the lipid extract was again dissolved in 1 mL of chloroform and was used for lipid profiling (see below). The extracted seedling debris was dried in an oven at 105 °C overnight, cooled, and the dry weight was determined using a balance (Mettler Toledo ME104E, Mettler Toledo).

### 4.3. Lipid Profiling by MS

Lipids were profiled using automated electrospray ionization-tandem mass spectrometry to identify digalactosyldiacylglycerol (DGDG), monogalactosyldiacylglycerol (MGDG), phosphatidylglycerol (PG), phosphatidylcholine (PC), phosphatidylethanolamine (PE), phosphatidylinositol (PI), phosphatidylserine (PS), phosphatidic acid (PA), lysophosphatidylethanolamine (LPE), and lysophosphatidylcholine (LPC) head group classes. The lipids in each head group class were quantified in comparison with internal standards of that class. To ensure consistency for data for each molecular species throughout the mass spectral data acquisition periods, quality control (QC) samples were prepared by pooling an aliquot from each lipid sample and were analyzed recurrently among the experimental samples [68,84]. The intensity of each lipid species in the experimental samples was normalized using the QC analyte intensities [68]. To maintain data quality, lipid analytes for which either the amount (normalized mass spectral signal) per milligram of leaf dry weight was less than the limit of detection, or which had a coefficient of variation (standard deviation divided by mean of the amount of the analyte in the QC samples) greater than 0.3 were removed from the data set. The lipid values were reported as normalized intensity per mg leaf dry weight (Supplementary Materials Tables S1 and S2).

### 4.4. Statistical Analysis

We performed differential analyses on the lipidomics dataset by conducting three types of tests: (1) comparison between genotypes with a given treatment; (2) treatment effect test when the genotype is fixed; and (3) interaction effect test between a genotype and a treatment. Raw lipidomics data were transformed by using

$$\log_{2}\;\left(\frac{r_{\mathrm{ij}}+\;\delta }{R_{j}}\times 10^{6}\right)$$

where 𝑟_ij_ is the lipidomics data for the 𝑖th lipid in the 𝑗th sample, δ is the half of the smallest non-zero measure, and 𝑅_j_ is the upper quartile for sample 𝑗. The transformed data was analyzed with the voom function in the R package limma (https://bioconductor.org/packages/release/bioc/html/limma.html (accessed on 17 February 2022)). A linear model was fit to each lipid and an empirical Bayes moderated F-statistic was calculated for testing differential expression. To account for multiple testing effects, all *p*-values were converted to q-values [85]. The resulting q-values were compared to a significance level of 0.05 to control the false discovery rate around 5%. Lipids with q-values no larger than 0.1 were declared to be statistically different.

A principal component analysis (PCA) was performed using the pcomp function of the factoextra package in R. Clustering for heat maps was performed using the pheatmap package in R. Spearman’s correlation scatter plot was generated using the ggplot2 package in R.

### 4.5. Unsaturation Index

The unsaturation index for a lipid molecular species was calculated as the average number of double bonds per acyl chain, which is the number of double bonds in the lipid molecular species divided by the number of acyl chains. The unsaturation index of a lipid headgroup class was calculated as previously described [52].

### 4.6. MALDI Mass Spectrometry Imaging

Leaves were placed on packing tape and dried under vacuum for about 1 h. Once dry, the tape was folded, enclosing the leaf between the tape, and passed through a rolling press. The tape pieces were pulled apart, resulting in the fracturing of the leaf to expose internal metabolites [52]. The tape pieces were aligned on glass slides for analysis. Matrices were applied as follows: 2,5-dihydroxybenzoic acid (DHB) was sublimated for 5 min at 140 °C and the binary matrix DHB: Fe_3_O_4_ (75 mm:5 mm in IPA) was sprayed using an oscillating capillary nebulizer (OCN) [86]. Negative mode data were acquired using 1,5-diaminonaphthalene (DAN). Data were acquired using a MALDI-linear ion trap (LIT)-Orbitrap mass spectrometer (MALDI-LTQ-Orbitrap Discovery; Thermo Finnigan, San Jose, CA, USA) with an external frequency-tripled Nd: YAG laser (UVFQ; Elforlight Ltd., Daventry, UK). The laser energy was optimized for each matrix and ten laser shots were used for every raster step. A raster step of 100 μm was used for imaging the fractured leaves. All images shown were generated using ImageQuest software version 1.1.0 (Thermo Scientific; San Jose, CA, USA). A mass tolerance of ±0.01 Da was used to produce MS images at each pixel. The maximum and minimum values for a particular metabolite in all sample types were set to the same values to facilitate comparisons. Peak assignments were made using accurate mass.

## Figures and Tables

**Table 1:** Mutants used in this study.

Genotype	Gene Function	Autophagy Phenotype	References
*atg5-1*	Required for formation of ATG8-PE conjugate via a ubiquitin like conjugation system	Autophagy blocked under all conditions tested	[26,32]
*atg7-2*	Required for formation of ATG8-PE conjugate via a ubiquitin like conjugation system	Autophagy blocked under all conditions tested	[33,34,35]
*atg9-4*	Lipid scramblase required for autophagosome formation	Autophagy blocked under all conditions tested (phenotype is less severe than *atg5* and *atg7*)	[12]
*kin10*	Catalytic subunit of the protein kinase SnRK1, activated by low energy	Autophagy activation by stress blocked	[11]
*raptor1b*	Subunit of the TOR complex, inactivated by low nutrients	Constitutive autophagy	[36]
*rns2-2*	Vacuolar ribonuclease	Constitutive autophagy	[12]
*rns2-2*; *atg5-1*	Double mutant	Autophagy blocked under all conditions tested	[12]
*rns2-2*; *atg9-4*	Double mutant	Autophagy blocked under all conditions tested	[12]

## Data Availability

The data presented in this study are available within the article and Supplementary Materials.

## Supplementary Materials

The following are available online at https://www.mdpi.com/2218-1989/12/2/190/s1. Figure S1: Nitrogen starvation alters lipid composition of *Arabidopsis* seedlings, Figure S2: Lipid changes in autophagy-altered mutants under +N conditions, Figure S3: Changes in polyunsaturated and very long chain fatty acids in autophagy-blocked mutants compared to WT under +N conditions, Table S1: Lipid amounts (nmol/mg dry weight), Table S2: Normalized average amount of each lipid species for each genotype under +N and −N conditions.
