# Correction: Mugume et al. Complex Changes in Membrane Lipids Associated with the Modification of Autophagy in *Arabidopsis*. *Metabolites* 2022, *12*, 190

**DOI:** 10.3390/metabo12090810

**Published:** 2022-08-29

**Authors:** Yosia Mugume, Geng Ding, Maria Emilia Dueñas, Meiling Liu, Young-Jin Lee, Basil J. Nikolau, Diane C. Bassham

**Affiliations:** 1 Department of Genetics, Development and Cell Biology, Iowa State University, Ames, IA 50011, USA; ymugume@iastate.edu; 2 Roy J. Carver Department of Biochemistry, Biophysics and Molecular Biology, Iowa State University, Ames, IA 50011, USA; gengding@iastate.edu (G.D.); dimmas@iastate.edu (B.J.N.); 3 Department of Chemistry, Iowa State University, Ames, IA 50011, USA; maria.duenas@newcastle.ac.uk (M.E.D.); yjlee@iastate.edu (Y.-J.L.); 4 Department of Statistics, Iowa State University, Ames, IA 50011, USA; mliu@fredhutch.org; 5 Public Health Sciences Division, Fred Hutchinson Cancer Research Center, Seattle, WA 98109, USA; 6 Center for Metabolic Biology, Iowa State University, Ames, IA 50011, USA

## Equation Correction

In the original article [1], there was a typographical error in the equation in Section 4.4: Statistical Analysis. The correct equation is as follows:

$$\log_{2}\left(\frac{r_{\mathrm{ij}}+\;\delta }{R_{j}}\times 10^{6}\right)$$

The authors apologize for any inconvenience caused and state that the scientific conclusions are unaffected. This correction was approved by the Academic Editor. The original publication has also been updated.
