# Supplementary material for: Complex Changes in Membrane Lipids Associated with the Modification of Autophagy in Arabidopsis
Source: Metabolites. 2022 Feb 18;12(2):190. doi: 10.3390/metabo12020190 (PMC8876039; doi:10.3390/metabo12020190)
Supplement: Supplementary file 1 [file metabolites-12-00190-s001.zip › metabolites-1530731-Supplementary Figures and legends.pdf]

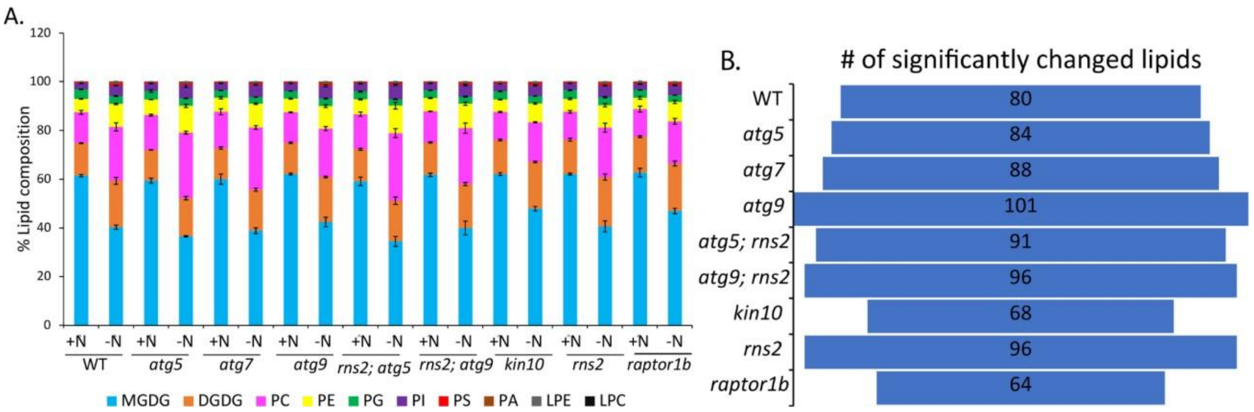

Supplementary Figure S1. Nitrogen starvation alters lipid composition of Arabidopsis seedlings. (A) Composition of galactolipids and phospholipids as a percentage of total lipids is shown. Percentage composition of each head group was calculated from means of individual lipid species. Data represent mean + SD of 3 replicates. (B) Genotypes respond to N starvation differently. The bar plot displays the set number of significantly changed lipids in each of the mutants and the WT.

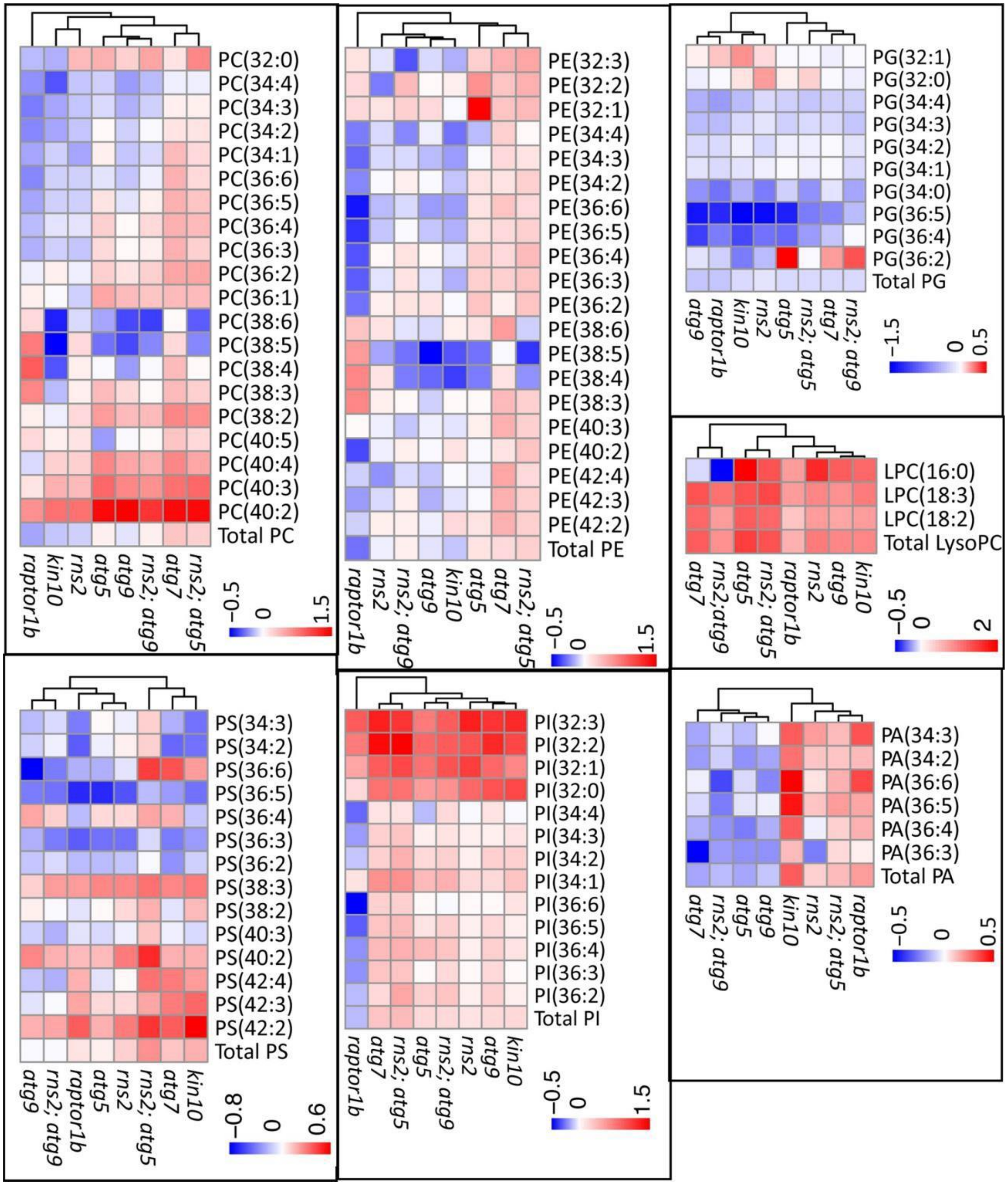

Supplementary Figure S2. Lipid changes in autophagy-altered mutants in +N conditions. Log$_2$ fold changes between mutants and WT are shown as heat maps with shades of red or blue according to the scale bar. The lipids represented on the heat maps are phosphatidylethanolamine (PE), phosphatidylcholine (PC), phosphatidylinositol (PI), phosphatidylglycerol (PG), phosphatidylserine (PS), lysophosphatidycholine (LPC) and phosphatidic acid (PA).

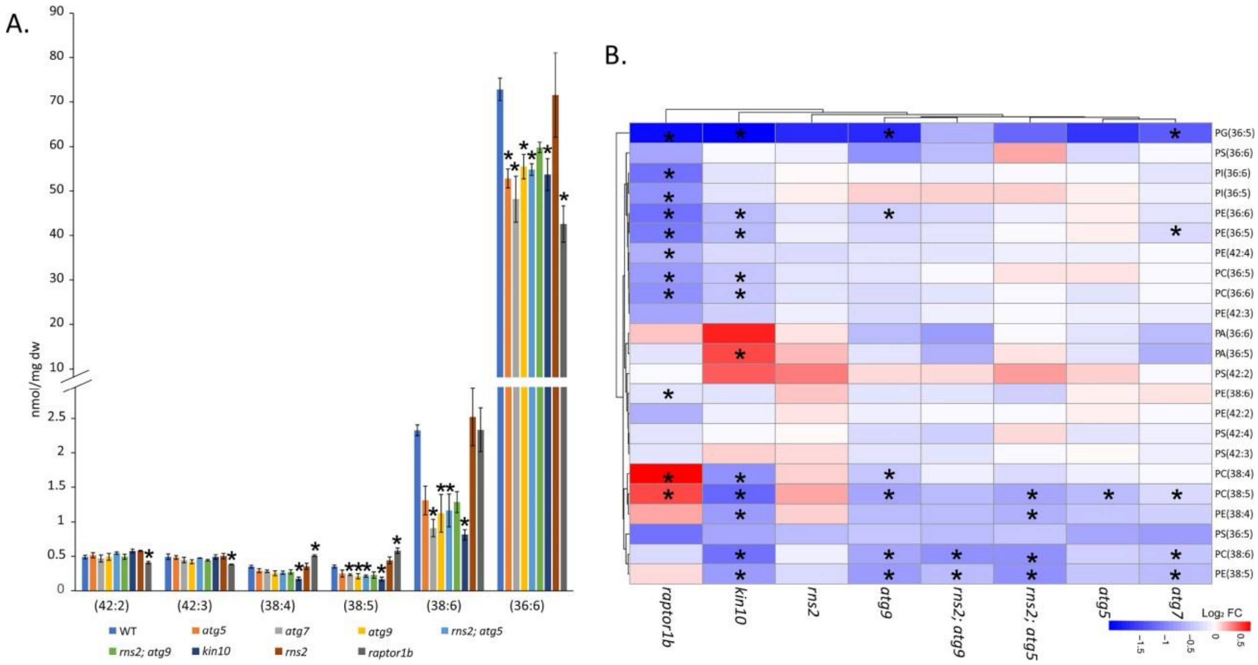

Supplementary Figure S3. Changes in polyunsaturated and very long chain fatty acids in autophagy-blocked mutants compared to WT in +N conditions (A). Changes in lipid amounts among lipid groups. Bar graph represents the amount of lipids per group based on acyl chain length and number of double bonds. Groups represent a summation of all lipid species from all polar head groups with the same chain length and number of double bonds. The values are the mean ± SD (n = 3 biologically independent samples, for WT and *atg5* n = 2 biologically independent samples). * denotes statistically significant differences between means (q < 0.1). (B) Heat map depicting $\log_2$-fold changes in individual lipid species that make up the lipid groups in mutants compared with WT under +N conditions. * denotes statistically significant differences (q < 0.1). Clustering for heat maps was performed using pheatmap package in R (https://cran.r project.org/web/packages/pheatmap/pheatmap.pdf).
